# A New Health Promotion Program That Includes Wadaiko Rhythm Exercise to Maintain the Health of Persons Excluded from Receiving Specific Health Guidance

**DOI:** 10.3390/ijerph19148520

**Published:** 2022-07-12

**Authors:** Suzumi Okuda, Miwako Tunematsu, Keiji Tabuchi, Toshio Kobayashi, Masayuki Kakehashi, Hisae Nakatani

**Affiliations:** 1 Graduate School of Biomedical and Health Sciences, Hiroshima University, 1-2-3, Kasumi, Minami-ku, Hiroshima 734-8553, Japan; tsunematsu@hiroshima-u.ac.jp (M.T.); kakehashi@hiroshima-u.ac.jp (M.K.); hinakata@hiroshima-u.ac.jp (H.N.); 2 Nursing Science Unit, Research and Education Faculty, Medical Sciences Cluster, Kochi University, 185-1, Kohasu Oko-cyo, Nankoku-City 783-8505, Japan; tabuchi@kochi-u.ac.jp; 3 School of Nursing and Health, Aichi Prefectural University, Tohgoku, Kamishidami, Moriyama-ku, Nagoya-City 463-8502, Japan; tkobaya@nrs.aichi-pu.ac.jp

**Keywords:** lifestyle habits, health promotion, Wadaiko rhythm exercise, specific health guidance inapplicable individuals, health behavior

## Abstract

This study examined the effectiveness of a new health promotion program, which is a collective intervention program aimed at improving lifestyle habits. It was designed as a one-year prospective cohort study. This program targeted non-obese persons at risk of developing lifestyle-related diseases and participants with hypertension, dyslipidemia, or hyperglycemia who were not included in the specific health guidance system in Japan. The Wadaiko rhythm exercise, which is a traditional performing art, is incorporated into this intervention as an enjoyable routine that can help participants continue the program, preventing them from dropping out. After a one-year follow-up, the effectiveness of the health promotion program was evaluated in 18 participants (2 males, 16 females; mean age 65.2 ± 3.4 years) and 92 controls. The results showed that triglyceride in the intervention group significantly decreased (−24.5 mg/dL; *p* = 0.02; 95% confidence interval [CI], −44.73 to −4.27) and high-density lipoprotein cholesterol significantly increased (+6.1 mg/dL; *p* < 0.01; 95% CI, 2.46 to 9.65), although levels in the control group did not change. These results suggest that the health promotion program could contribute to lifestyle habit improvements in those who are excluded from receiving specific health guidance.

## 1. Introduction

Japan has one of the highest life expectancies in the world [1]. However, there are concerns about increasing lifestyle-related diseases owing to rapid aging and diversification of lifestyle habits. Lifestyle-related diseases, such as cancer and cardiovascular diseases, account for 60% of the causes of death, and the worsening of lifestyle-related diseases leads to a decline in quality of life. These are major issues in Japan [1]. In 2000, the philosophy of the WHO’s health promotion was adopted in Health Japan 21 to respond to the aging population and the increase in lifestyle-related diseases [2]. This measure focused on individual lifestyles with an emphasis on primary prevention, but the social environment was scarce [1]. In 2013, Health Japan 21 (the second term) indicated that quality of life could be improved by preventing both the onset and worsening of lifestyle-related diseases through the improvement of individual lifestyles and the social environment surrounding them [1]. Against the background of such changes in health promotion measures, a specific health checkup/health guidance system unique to Japan was introduced to improve the lifestyles of individuals with multiple risk factors for lifestyle-related diseases. However, this system only targets obese persons; therefore, non-obese persons and persons using medications for cardiovascular disease or diabetes were excluded [3]. Previous reports suggest that the risk and prevalence of stroke are higher in the non-obese group with the risk of lifestyle-related disease than in the obese group; thus, interventions among non-obese individuals to prevent lifestyle-related diseases are necessary [4,5]. Furthermore, interventions to improve lifestyle habits are beneficial for patients with lifestyle-related diseases who use medications [6,7]. Therefore, it is critically important to improve the habits of non-obese persons who are at a high risk of lifestyle-related diseases and persons who are using medications; thus, it is necessary to provide opportunities for them to receive health guidance. Providing opportunities is one of the essential activities of health promotion [2]. Even for those who are excluded from the health guidance system, it is necessary to provide opportunities to increase their personal empowerment so that they can improve their relevant habits.

Exercise is known to be useful for improving lifestyle habits [8,9,10]. Nevertheless, the percentage of Japanese who habitually exercise is as low as 31.7%, and there has not been a major change in the percentage over the past decade [11]. These findings suggest that it is difficult to change poor exercise habits. Interestingly, physical activity through social participation in hobbies or sports organizations has been shown to be more effective than personal exercise [12,13]. Thus, these studies suggest that collective interventions are more effective for behavior modification than individual interventions. Moreover, those excluded from receiving specific health guidance are left without intervention, although they may have a considerably similar risk to that of those who are included. Therefore, we conducted a health promotion program through collective intervention for persons excluded from a specific health guidance system. Since the health promotion program was implemented for 7 months, it was necessary to prevent participants from dropping out. The key to preventing dropouts is to make the participants enjoy the program, as well as form friendships and engage in social communication [14,15]. Therefore, we adopted the Wadaiko rhythm exercise, which combines exercise and traditional performing arts. This exercise used songs that were popular in the community. It did not use Wadaiko itself. Therefore, it is an actionable tool that is easily accepted in the community. There are few studies on Wadaiko related to health [16,17] or studies incorporating traditional performing arts in health promotion [18]. In addition, studies as late as approximately 2014 have reported on the effects of specific health guidance [19], but to our knowledge, they have not reported on the effects of group interventions for people who are excluded from specific health guidance at all.

The objective of this study was to examine the hypothesis that a health promotion program incorporating traditional performing arts improves the lifestyles of those who are excluded from specific health guidance.

## 2. Materials and Methods

### 2.1. Outline of the Town Surveyed and Selection of Participants

This health promotion program was conducted on an isolated island located in the inland sea of Japan. The population aging rate (≥65 years) was 47.2%, which is one of the highest in Japan [20]. Regarding the selection of target persons, the isolated town conducted a public offering with priority given to individuals aged ≤70 years (*n* = 209) who were excluded from receiving specific health guidance though requiring it. The target selection criteria were the presence of hypertension (systolic blood pressure ≥130 mmHg or diastolic blood pressure ≥ 85 mmHg), dyslipidemia (triglyceride (TG) ≥150 mg/dL or low-density lipoprotein cholesterol (LDL-C) ≥120 mg/dL or high-density lipoprotein cholesterol (HDL-C) <40 mg/dL), and hyperglycemia (hemoglobin A1c (HbA1c) ≥5.6% or fasting plasma glucose ≥100 mg/dL). However, those who were subjected to specific health guidance were excluded. Twenty-two participants voluntarily applied for the program and obtained physician clearance before participating. They were followed-up for 1 year, starting from August 2014 (observation period: August 2014 to October 2015). The participants who completed the tracking were defined as the intervention group (participant group). For the control group (non-participant group), persons who underwent specific health checkups 1 year later and provided consent for the study were selected from among 187 participants aged ≤70 years who did not participate in the health promotion program. We decided not to perform matching in an effort to use as much data as possible for those in the control group who agreed to participate in the study.

### 2.2. Structure of Health Promotion Program

The health promotion program was conducted for 7 months, from August 2014 to February 2015. The program consisted of 14 sessions in total, each lasting for 2.5 h, including health and nutrition lectures, group exercises, individual interviews, and group meetings. In health lectures, physicians addressed lifestyle-related diseases. In the nutrition lectures, nutrition experts explained what to eat and, using food replicas, how to prepare healthy lunch boxes. Moreover, participants received instructions on how to review their eating behaviors. In individual interviews and group meetings, public health nurses provided participants with feedback on the blood test results shown in the figures and tables, encouraging them to make changes to their behavior. The group exercise activities by health fitness programmers included warm-ups, aerobics, resistance, and cool-down exercises. The intensity of exercise recommended for patients with lifestyle-related diseases ranged from “fairly light” to “somewhat hard,” using the Borg scale level [21]. We adopted the Wadaiko rhythm exercise 3 months after starting the health promotion program to prevent participants from dropping out. The Wadaiko rhythm exercise included strength training, aerobic, and balance exercises. The songs used in the Wadaiko rhythm exercise are performed at events and festivals in the town and are popular in the community. Although this Wadaiko rhythm exercise did not involve the use of Wadaiko, participants behaved as if they were holding drumsticks and hitting the Wadaiko, coordinated with the sound of the Wadaiko. Participants made a loud sound, “Yeah,” while performing. The exercise intensity was measured as 60–70% of the heart rate calculated for age (220-age, i.e., 220 minus age), the Borg scale level 10–13 of the rate of the perceived exertion, and a music tempo of 126 beats per min. A session of exercise lasted for approximately 6 min and was performed twice. After the health promotion program was complete, 18 out of the 22 participants voluntarily formed a group to continue group exercise activities once a month.

### 2.3. Evaluation Items and Data Collection

The following items were evaluated: blood lipid levels, blood glucose levels, exercise habits, medication history, medical history, and smoking history [3]. In addition, the participants completed a questionnaire on the Wadaiko rhythm exercise. TG, HDL-C, LDL-C, and HbA1c levels were determined through blood testing. Blood testing of the intervention group was conducted at a medical institution commissioned by the town. Regarding exercise habits, we asked for a “yes” or “no” answer as to whether they actively exercised for >30 min each time, twice weekly for over a year. We requested a response regarding the Wadaiko rhythm exercise using free text: “How did you feel when you performed the Wadaiko rhythm exercise?” and “How did you feel about the rhythmical momentary shout that you made while performing the Wadaiko rhythm exercise?” Medication, medical, and smoking histories were obtained from the results of specific health checkups. All data from the control group were obtained from the results of specific health checkups. Regarding exercise habits in the control group, a “yes” or “no” answer was required regarding whether they were exercising and lightly sweating for >30 min each time, twice weekly for over a year [3]. Smoking history in both groups consisted of three categories: never smoked, smoked in the past (former smokers), and currently smoking (smokers).

### 2.4. Design of the Intervention Program

This was a one-year prospective cohort study. We conducted a health promotion program for individuals aged >50 years and ≤70 years who were excluded from receiving specific health guidance, and we examined the effects of the program. Our participants were non-obese persons and persons using medicines for cardiovascular disease or diabetes who were excluded from receiving health guidance [3]. A control group was used for comparison. To prevent dropouts, we adopted the Wadaiko rhythm exercise as part of the health promotion program. The evaluation indices were blood lipid and blood glucose levels. In addition, we administered a questionnaire on Wadaiko rhythm exercises to the participants. The survey was conducted at the beginning of the health promotion program period and 1 year later. In this evaluation, we first analyzed the changes in blood lipid and blood glucose levels. Next, the factors that influenced the effects were analyzed using the data from the changes. The Wadaiko rhythm exercise questionnaire was used to evaluate the feelings of enjoyment that are important for the continuation of the health promotion program.

### 2.5. Statistical Analysis

The baseline values of the two groups were compared using the unpaired t-test and Mann–Whitney U test for continuous variables and Fisher’s exact test for discrete variables. Paired data showing progress after 1 year were analyzed using the paired t-test for normally distributed variables and the Wilcoxon signed-rank test for non-normally distributed variables. In the comparison of the differences between the two groups, normally distributed variables were analyzed using the unpaired t-test, and non-normally distributed variables were analyzed using the Mann–Whitney U test. The factors influencing these differences were evaluated using multiple regression analysis. Regression analysis was performed with the simultaneous forced entry of age, sex, BMI, exercise habits, participation in health promotion programs, smoking, former smoking, use of cholesterol-reducing drugs, antihypertensive drugs, insulin injection, or antihyperglycemic drugs as independent variables. The 3 choices of smoking status were converted into 2 dichotomous variables: the smoker variable was 1 only among smokers, whereas the former smoker variable was 1 only among former smokers. Independent variables were selected based on previous studies and questionnaires for specific health checkups. Changes in TG, HDL-C, LDL-C, and HbA1c were chosen as dependent variables of linear regression models. Stepwise regression, according to *p*-values, was used as the variable selection method. All statistical analyses were performed using EZR version 1.54 [22] with a significance level of 5%. Normality of distribution was only rejected when both the Kolmogorov–Smirnov, and Shapiro–Wilk normality tests had *p* < 0.05, for uniformity and simplicity of analyses. The free description of the impression of the Wadaiko rhythm exercise was categorized based on the similarity of the semantic and descriptive content.

## 3. Results

### 3.1. Workflow of Our Research

**Figure 1:**
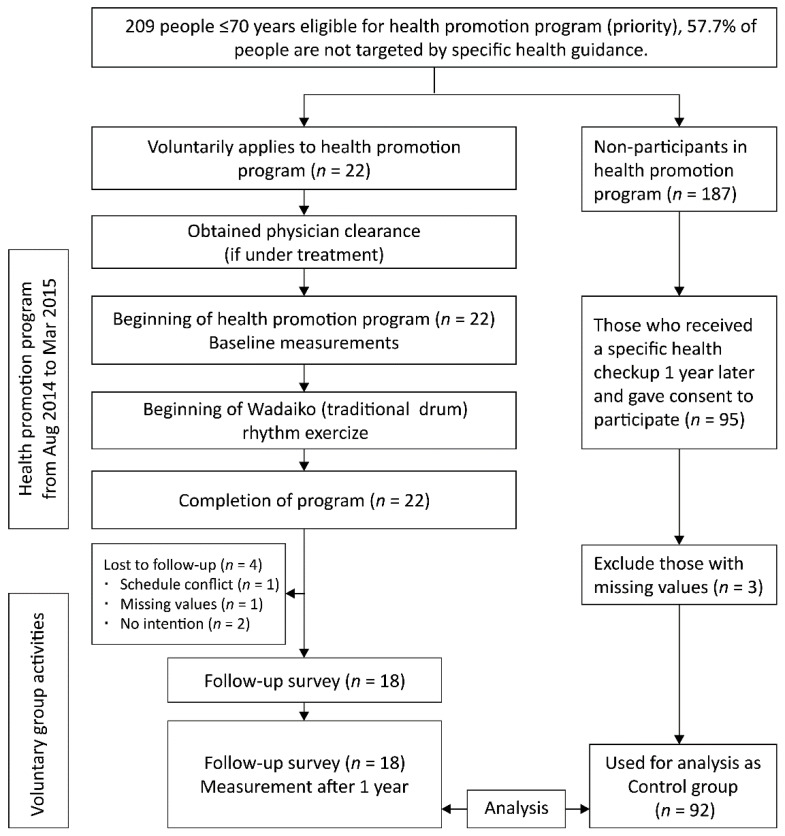
Study workflow diagram showing inclusion and exclusion criteria.

Figure 1 shows the workflow of this study. Twenty-two persons applied for and completed the health promotion program. Although the participation continuation rate of the health promotion program was 100%, the intervention group was composed of 18 persons (2 males, 16 females; mean age 65.2 ± 3.4 years) who received follow-up surveys for 1 year. Of the 187 people who did not participate in the health promotion program, 95 underwent a specific health checkup and provided informed consent 1 year later. A total of 3 persons with missing values were excluded from the study, and the remaining 92 individuals (33 males, 59 females; mean age 64.1 ± 4.5 years) were used as the control group.

### 3.2. Baseline Measurements

Table 1 shows the baseline characteristics of the participants. HbA1c in the intervention group was higher than that in the control group (5.87 ± 0.40% vs. 5.61 ± 0.46%, *p* < 0.01), and the proportion of participants receiving insulin injections or antihyperglycemic drugs in the intervention group was also higher than that in the control group; however, there were no other significant differences between the study groups.

### 3.3. Changes in Blood Measurements

Table 2 shows changes observed during the one-year follow-up in the intervention and control groups. The intervention group had a significant decrease in TG level after 1 year (*p* = 0.02; 95% CI, −44.73 to −4.27) and a significant increase in HDL-C level (*p* < 0.01; 95% CI, 2.46 to 9.65). However, the control group showed a significant decrease in the HDL-C level (*p* = 0.02; 95% CI, −3.19 to −0.22). Although the LDL-C in the intervention group did not decrease significantly, the LDL-C corresponding to the level of “visiting hospitals” (≥140) at the baseline improved to the level of “receiving health guidance” (≥120). The median HbA1c level in the control group was not lower than the level of “receiving health guidance” (≥5.6), but it showed a significant improvement (*p* = 0.04) after 1 year. The intervention group showed a non-significant decrease. Table 3 shows a comparison of changes in the two groups. A considerable difference in all results was observed between groups. TG significantly decreased in the intervention group (*p* = 0.02; 95% CI, −46.10 to −5.05). HDL-C significantly increased in the intervention group (*p* < 0.01; 95% CI, 4.10 to 11.42), whereas HDL-C significantly decreased (worsened) in the control group.

### 3.4. The Influential Factors of Change

Table 4 shows the partial regression coefficients of the factors that influenced the changes in TG, HDL-C, LDL-C, and HbA1c levels. Stepwise multiple regression analysis using *p*-values showed that the health promotion program remained in the final model of TG and HDL-C.

TG was not very influential but significantly decreased by 25.58 mg/dL after participating in the health promotion program (R^2^ = 0.053, *p* = 0.015). HDL-C was also not very influential but significantly increased by 7.76 mg/dL (R^2^ = 0.14, *p* < 0.001). The variables selected in the final model produced the same results as the variables that showed significant values prior to the variable selection. LDL-C level had no variables in the final model. Logarithmic transformation for HbA1c was performed to achieve a normal distribution; however, HbA1c was not normally distributed. HbA1c levels significantly increased in users of insulin injections or antihyperglycemic drugs (R^2^ = 0.053, *p* = 0.016). Participation in the health promotion program was the most influential factor in improving the TG and HDL-C levels. All models had a variance inflation factor (VIF) that ranged from 1.06 to 3.16.

### 3.5. Impressions of the Wadaiko Rhythm Exercise

The evaluation of the Wadaiko rhythm exercise by participants showed that 11 persons (61.1%) described the enjoyment provided by shouting with friends and moving rhythmically. In addition to having enjoyment, they felt positive effects on stress coping and a sense of solidarity from the shouts that were characteristic of the Wadaiko rhythm exercise.

## 4. Discussion

The effect of the health promotion program was evaluated based on the change in blood test data after 1 year. In comparing the intervention and control groups, a significant improvement in TG and HDL-C levels was observed in the intervention group. These results suggest that participation in health promotion programs is an influential factor in these changes. In addition, the Wadaiko Rhythm Exercise, using traditional performing arts, had positive effects on participants, such as having a sense of enjoyment, solidarity, and better coping with stress.

### 4.1. Influential Elements in the Health Promotion Program

Three elements of the health promotion program were considered to facilitate the improvement of lifestyle habits.

The first element was the adoption of the Wadaiko rhythm exercise. The results of the questionnaire survey showed that the participants enjoyed the Wadaiko rhythm exercise as expected. In addition, participants benefited from the characteristic shouts of the Wadaiko exercise; they reported having fun, a sense of solidarity, and better coping with stress. Therefore, the participants did not drop out, resulting in a 100% retention rate. The health promotion program included exercise. Since it is usually difficult to continue exercising [23,24], it is necessary to prevent the participants from dropping out. The program should include a sense of enjoyment, friendship, and social communication, which have been reported as continuity factors [14,15]. People over the age of 60 tend to meet, talk, and interact with close friends and like-minded people as daily sources of pleasure [25]. Therefore, we introduced the Wadaiko performance, which was known to the community. The Wadaiko performance may be associated with improved psychological stress, and the rhythmic cooperative behavior observed in people engaging in Wadaiko may improve not only physical health but also communication skills [16,17]. Studies on drums in other countries have also reported psychological effects that cause relaxation, enjoyable experiences, and a sense of connection [26]. These results were consistent with the results of our Wadaiko rhythm exercise. It can be considered that the emotions caused by the Wadaiko rhythm exercise prevented the participants from dropping out. In addition, a six-month lifestyle intervention study by several experts similar to our study showed many positive changes, but no changes in lipid metabolism and blood glucose levels were reported [27]. Another aspect that is specific to our study may be the sense of enjoyment. The emotion of enjoyment has a positive effect on a person’s exercise [28]. Moreover, we used popular songs that were familiar to all participants alike in the Wadaiko rhythm exercise. It elicited feelings of enjoyment. Creating an environment that promotes such positive feelings toward exercise is a kind of health promotion strategy [28]. It can be considered that this environment supported individual empowerment and led to improvements in lifestyle habits and blood measurement data. There was another successful health promotion program making use of traditional culture to induce motivation. In the health promotion program, a modified version of Awa Odori, a traditional mass-gathering dance in the Awa district, was involved in the exercise [18]. This exercise was reported to spread in the community because Awa Odori was very familiar in the community. We believe and hope it is likely that our Wadaiko rhythm exercise will spread. Our Wadaiko rhythm exercise did not involve the use of Wadaiko itself, but similar to the actual Wadaiko performance, participants behaved as if they were holding drumsticks and hitting the Wadaiko, coordinated with a song and the sound of Wadaiko. They loudly shouted “Yah” several times while performing. Such shouts are characteristic of the Wadaiko performance. They performed these actions altogether. Furthermore, the song was one of the popular songs in the community. Wadaiko rhythm exercise is (in a sense) a virtual Wadaiko performance that realizes the advantages of using traditional performing arts in the exercise program.

The second aspect is professional education. Lectures and consultations were provided by a medical doctor and a nutritionist in our program. The TG level of the intervention group significantly decreased (−24.5 mg/dL; *p* = 0.02; 95% CI, −44.73 to −4.27), and the HDL-C level significantly increased (+6.1 mg/dL; *p* < 0.01; 95% CI, 2.46 to 9.65). Nutritional education for elderly women has been reported to improve dietary habits and nutrition, leading to an increase in blood HDL-C levels [29,30]. Furthermore, multimedia education combined with nutrition therapy has been shown to improve HbA1c and TG levels in type 2 diabetes [31]. Similar to these reports, our multidisciplinary professional education may have contributed to the improvement in TG and HDL-C levels through the improvement of lifestyle habits.

The third aspect is data visualization. The public health nurse provided the results of the blood test to the participants as figures and tables so that they could easily understand the improvement. Participants who received such feedback noticed the changes that had occurred in their bodies and were given an opportunity to reconsider their behavior. It has been shown that recognition of facts changes eating habits [32]. Since behavioral changes due to recognition are not restricted to eating behavior, it was considered that data visualization also contributed to the improvement of lifestyle habits.

### 4.2. Decrease in LDL-C without Specific Influential Factors

Both groups showed decreases in LDL-C levels after 1 year, although this was not statistically significant. The intervention group particularly improved from the level of “visiting hospitals” to the level of “receiving health guidance.” However, no factor affecting this decrease was identified. Exercise [8,9] and dietary effects are known to improve LDL-C levels. The increase in fiber intake was reported to be beneficial, although the type and duration were unknown, and soluble fiber has been reported to reduce LDL-C levels [33,34]. A larger decrease in LDL-C levels was observed in the intervention group than in the control group. This may be due to the effects of one or more of the observed factors of the health promotion program.

### 4.3. HbA1c among Antihyperglycemic Drug Users

Analysis of the changes in HbA1c levels from baseline to the one-year follow-up showed a significant decrease in the control group, but not in the intervention group (HbA1c reflects the average blood glucose level in the preceding 1–2 months). It must be noted that the HbA1c level after 1 year increased among people using insulin injections or antihyperglycemic drugs. A similar result was reported in a study investigating HbA1c levels during the Chinese New Year. The authors concluded that the winter holiday season, and the accompanying opportunities for dinners, may pose a special risk to people with long-standing type 2 diabetes [35]. Although this survey study period did not cover winter holidays, Obon, a traditional summer holiday in Japan, may have affected glycemic control. The intervention group had a higher proportion of insulin injection or antihyperglycemic drug users, as well as higher baseline HbA1c levels than the control group. This could explain why HbA1c levels in the intervention group did not decrease. Therefore, it should be remarked that insulin deficiency and the delay in the recovery of blood glucose levels thereafter might have been caused by overeating, lack of exercise, and stress during a special holiday period in patients with type 2 diabetes.

Our results suggest that non-obese persons and those who were using medication for cardiovascular disease or diabetes who were excluded from receiving specific health guidance should participate in a health promotion program that integrates health promotion and traditional performing arts. Health promotion programs may contribute to the improvement of lifestyle habits. Additionally, our strategy seems to be effective for persons who cannot get into the habit of exercising for more than 30 min twice weekly. Unfortunately, the COVID-19 pandemic has placed restrictions on people’s behavior. Physical distance affected the feeling of loneliness, and the decrease in physical activity affected interaction with friends and social participation [36,37], changing people’s lifestyles. Web-based activities are widely offered as a means of complementing this environment, but the elderly have challenges in terms of the lack of interest and technological literacy. However, it is worth noting that the participants appreciated the enjoyment of being with others [38]. Such enjoyment and sense of solidarity that our health promotion program can bring will play an important role in improving lifestyles that have changed due to long physical distances and less interaction with friends during the COVID-19 pandemic. One limitation of our study is that our participants volunteered to participate due to public introduction in their municipalities, which might have caused the findings to be biased. Furthermore, the municipality-constrained focus also led to a relatively small sample size that could not be increased because of the local government. In the future, it will be necessary to conduct similar health promotion programs in many communities, allowing a broader accumulation of data.

## 5. Conclusions

Our program was the first health promotion program implemented using traditional performing arts for people excluded from health guidance systems in Japan. A health promotion program conducted in the community showed beneficial effects on TG and HDL-C in non-obese persons and those using medication for cardiovascular disease or diabetes. In addition, the Wadaiko rhythm exercise showed positive effects, such as having a sense of enjoyment, solidarity, and coping with stress. Interventions that included this exercise contributed to the creation of a new environment for improving lifestyle habits. As far as we know, there is no other report on a health promotion program that involves such enjoyable traditional performing arts while demonstrating the importance of serving uncovered individuals who are at risk and excluded from guidance systems. Our findings suggest that a method that combines traditional performing art and health promotion may contribute to lifestyle improvements. This group program will likely play an important role in improving lifestyle habits, especially after the COVID-19 pandemic is over.

## Figures and Tables

**Table 1:** Baseline characteristics.

Characteristic	Intervention Group (*n* = 18)	Control Group (*n* = 92)	*p* Value
Age, years (SD)	65.2 (3.4)	64.1 (4.5)	0.39
Sex, female, *n* (%)	16 (88.9)	59 (64.1)	0.05
Body mass index, kg/cm^2^ (SD)	23.6 (3.6)	22.1 (2.6)	0.21
TG, mg/dL (SD)	112.6 (39.4)	105.2 (48.6)	0.21
HDL-C, mg/dL (SD)	64.8 (14.3)	66.1 (15.5)	0.75
LDL-C, mg/dL (SD)	140.8 (30.5)	131.9 (29.5)	0.24
HbA1c, % (SD)	5.87 (0.40)	5.61 (0.46)	<0.01
Anti-hypertensive drugs, *n* (%) ^a^	8 (44.4)	24 (26.1)	0.16
Insulin injections or antihyperglycemic drugs, *n* (%) ^a^	4 (22.2)	5 (5.4)	0.04
Cholesterol-reducing drugs, *n* (%) ^a^	8 (44.4)	28 (30.4)	0.28
History of stroke, *n* (%)	0 (0.0)	3 (3.3)	1
History of heart disease, *n* (%)	2 (11.1)	3 (3.3)	0.19
History of chronic kidney failure, *n* (%)	0 (0.0)	0 (0.0)	—
History of smoking, *n* (%)	3 (16.7)	31 (33.7)	0.18
Exercise habits, *n* (%) ^b^	9 (50.0)	34 (37.0)	0.31

Abbreviation: TG, triglyceride; HDL-C, high-density lipoprotein cholesterol; LDL-C, low-density lipoprotein cholesterol; HbA1c, hemoglobin A1c. ^a^ Taking prescription drugs. ^b^ Those who responded “yes” had been exercising for over a year, at least twice a week, with each session lasting more than 30 min. For continuous variables, the t-test was performed for those with normality, while the Mann–Whitney U test was performed for those without normality. Fisher’s exact test was performed for the ratio.

**Table 2:** Blood measurements at baseline and after 1 year.

		Baseline	1 Year	*p* Value	95% CI	
					LL	UL
Intervention group (*n* = 18)	TG, mg/dL (SD)	112.61 (39.38)	88.11 (28.47)	0.02 *	−44.73	−4.27
		111.5 (91.25–121.25)	86.0 (68.75–112.5)	0.02 *		
	HDL-C, mg/dL (SD)	64.83 (14.31)	70.89 (14.66)	<0.01 **	2.46	9.65
		60.5 (56.25–75.50)	70 (64.00–78.25)	<0.01 **		
	LDL-C, mg/dL (SD)	140.83 (30.46)	129.06 (29.71)	0.13	−27.38	3.82
		140.5 (127–157.5)	130.5 (111.50–141.75)	0.21		
	HbA1c, % (SD)	5.87 (0.40)	5.82 (0.46)	0.41	−0.19	0.08
		5.8 (5.650–5.975)	5.75 (5.5–5.800)	0.07		
Control group (*n* = 92)	TG, mg/dL (SD)	105.17 (48.60)	106.25 (50.76)	0.80	−7.22	9.38
		93.0 (72.00–129.75)	95.5 (70.50–129.0)	0.97		
	HDL-C, mg/dL (SD)	66.12 (15.47)	64.41 (15.85)	0.02 *	−3.19	−0.22
		66.0 (55.75–74.25)	61 (53.75–74.00)	0.045		
	LDL-C, mg/dL (SD)	131.86 (29.54)	128.02 (27.79)	0.07	−7.98	0.31
		133.0 (110–152.0)	128.5 (109.75–145.00)	0.10		
	HbA1c, % (SD)	5.61 (0.46)	5.57 (0.51)	0.06	−0.07	0.00
		5.6 (5.375–5.800)	5.60 (5.3–5.725)	0.04 *		

Abbreviation: TG, triglyceride; HDL-C, high-density lipoprotein cholesterol; LDL-C, low-density lipoprotein cholesterol; HbA1c, hemoglobin A1c; CI, confidence interval; LL, lower limit; UL, upper limit; 95% CI, confidence interval of the difference between baseline and 1 year later. * *p* < 0.05, ** *p* < 0.01: Significant difference vs. baseline data. The upper row shows the mean and standard deviation, and the lower row shows the median and interquartile range.

**Table 3:** Two-group comparison of changes in blood measurement.

	Intervention Group (*n* = 18)	Control Group (*n* = 92)	*p* Value	95% CI	
				LL	UL
TG, mg/dL (SD)	−24.50 (40.68)	1.08 (40.08)	0.02 *	−46.10	−5.05
	−28.5 (−41–9.5)	0.0 (−24–20.5)	0.03 *		
HDL-C, mg/dL (SD)	6.06 (7.23)	−1.71 (7.16)	<0.01 **	4.10	11.42
	5.5 (1.75–10.75)	−1.0 (−6.00–3.00)	<0.01 **		
LDL-C, mg/dL (SD)	−11.78 (31.37)	−3.84 (20.00)	0.17	−19.27	3.39
	−5.5 (−15.50–7.75)	−2.0 (−13.25–5.50)	0.63		
HbA1c, %; (SD)	−0.06 (0.28)	−0.04 (0.18)	0.70	−0.120	0.081
	−0.1 (−0.2–0.0)	0.0 (−0.1–0.1)	0.18		

Abbreviation: TG, triglyceride; HDL-C, high-density lipoprotein cholesterol; LDL-C, low-density lipoprotein cholesterol; HbA1c, hemoglobin A1c; CI, confidence interval; LL, lower limit; UL, upper limit; 95% CI, confidence interval of the difference between groups. * *p* < 0.05, ** *p* < 0.01: Significant difference two-group comparison. The upper row shows the mean and standard deviation, and the lower row shows the median and interquartile range.

**Table 4:** Influential factors of change.

Variable	TG		HDL-C		LDL-C		HbA1c	
	Estimate	*p* Value	Estimate	*p* Value	Estimate	*p* Value	Estimate	*p* Value
(Intercept)	131.810	0.044 *	5.583	0.649	27.598	0.462	0.074	0.819
Sex	2.518	0.860	−2.786	0.302	4.878	0.553	−0.068	0.338
Age	−1.517	0.093	0.020	0.905	−0.603	0.245	−0.001	0.902
Body mass index	−2.172	0.166	−0.264	0.373	−0.142	0.875	0.000	0.985
Exercise habits	8.646	0.268	−0.473	0.748	5.637	0.211	−0.007	0.865
Health promotion program	−22.652	0.040 *	8.038	<0.001 ***	−7.784	0.217	−0.027	0.618
Former smokers	23.596	0.103	−3.005	0.270	8.844	0.288	−0.032	0.661
Smokers	20.572	0.303	−4.414	0.243	13.884	0.229	−0.049	0.625
Cholesterol-reducing drugs	8.511	0.339	0.319	0.849	4.750	0.355	−0.020	0.647
Anti-hypertensive drugs	−7.491	0.431	0.899	0.616	1.205	0.826	−0.069	0.147
Insulin injection or antihyperglycemic drugs	25.318	0.090	0.045	0.987	−0.892	0.917	0.208	0.006 **
Multiple R-squared	0.18		0.164		0.070		0.097	
Adjusted R-squared	0.097		0.080		−0.024		0.006	
F-statistic	2.168 on 10 and 99 DF		1.944 on 10 and 99 DF		0.7418 on 10 and 99 DF		1.069 on 10 and 99 DF	
*p*-value	0.026		0.048		0.684		0.394	
**Final model**								
(Intercept)	1.076	0.798	−1.707	0.024 *			−0.052	0.007 **
Health promotion program	−25.576	0.015 *	7.762	<0.001 ***	Excluded all			
Insulin injection or antihyperglycemic drugs							0.164	0.016 *
Multiple R-squared	0.053		0.140				0.053	
Adjusted R-squared	0.045		0.133				0.044	
F-statistic	6.101 on 1 and 108 DF		17.64 on 1 and 108 DF				6 on 1 and 108 DF	
*p*-value	0.015		< 0.001				0.016	

Abbreviation: TG, triglyceride; HDL-C, high-density lipoprotein cholesterol; LDL-C, low-density lipoprotein cholesterol; HbA1c, hemoglobin A1c. * *p* < 0.05, ** *p* < 0.01, *** *p* < 0.001: Significant difference.

## Data Availability

The data used in this study are available from the corresponding author upon request.
